# Viscoelastic Properties of the Chinese Fir (*Cunninghamia lanceolata*) during Moisture Sorption Processes Determined by Harmonic Tests

**DOI:** 10.3390/ma9121020

**Published:** 2016-12-17

**Authors:** Tianyi Zhan, Jianxiong Lu, Jiali Jiang, Hui Peng, Anxin Li, Jianmin Chang

**Affiliations:** 1 State Key Laboratory of Tree Genetics and Breeding, Research Institute of Wood Industry, Chinese Academy of Forestry, Beijing 100091, China; tyzhan@njfu.edu.cn (T.Z.); jianxiong@caf.ac.cn (J.L.); penghyx@126.com (H.P.); lianxin6928@126.com (A.L.); 2 College of Materials Science and Engineering, Nanjing Forestry University, Nanjing 210037, China; 3 College of Materials Science and Technology, Beijing Forestry University, Beijing 100083, China; cjianmin@bjfu.edu.cn

**Keywords:** Chinese fir, sorption, viscoelasticity, harmonic test, mode of RH increment, plasticization effect, unstable state

## Abstract

Measured by harmonic tests, the viscoelastic properties of Chinese fir during moisture sorption processes were examined under three relative humidity (RH) modes: RH_ramp_, RH_isohume_, and RH_step_. The stiffness decreased and damping increased as a function of the moisture content (MC), which is presumed to be the effect of plasticization and an unstable state. The increasing damping was associated with the breaking of hydrogen bonds and the formation of free volume within polymer networks. The changes of loss modulus ratio at 1 and 20 Hz, *E*″_1Hz_/*E*″_20Hz_, proved the changing trend of the unstable state. Higher ramping rates aggravated the unstable state at the RH_ramp_ period, and higher constant RH levels provided more recovery of the unstable state at the RH_isohume_ period. Changes of viscoelastic properties were associated with RH (varied or remained constant), and the application of Boltzmann’s superposition principle is a good approach to simulate viscoelasticity development.

## 1. Introduction

Thermo-hydro-mechanical (THM) treatment of wood, including compression, welding, molding, etc., is a promising eco-friendly method of wood processing technology [1,2] based on the combined treatments of wood by elevated temperature, moisture, and applied mechanical loading (static or dynamic loading). Under the static or dynamic loading, wood exhibits different viscoelastic behaviors. The viscoelasticity of wood is highly dependent on the organization or properties of wood polymers [3,4]. The dynamic mechanical analysis (DMA) is a well-established rheological test that offers insight into the viscoelastic behavior of wood. The improved understanding of viscoelasticity could increase the efficiency of THM treatment and the quality of THM-treated wood. In addition, the development of new processes and products that are in relation to the viscoelastic nature of wood will be achieved [5].

The mechanical properties of wood, from linearly elastic to viscous, are dependent on the temperature, moisture content (MC), and time scale of the test [6,7,8,9]. MC influences nearly all the physical and mechanical properties of wood, as water plays a role of plasticizer, and it forms hydrogen bonds (HBs) to lignin, hemicelluloses, and paracrystalline cellulose. Thus the existing HBs within the polymers are substituted and decrease the wood’s stiffness [10]. The plasticization effect of moisture lowers the energy required to initiate chain mobility, resulting in a decrease in the glass transition temperature [11]. The effects of moisture are dependent on species, density, extractive content, and microstructure, and are additionally influenced by heating history, ambient temperature, and relative humidity (RH). Wood and wood-based products are subjected to a variety of RH conditions during processing, storage, and end use periods. The response of viscoelastic behavior to RH variation is referred to as the mechano-sorptive (MS) effect [12,13]. The RH variation provides MC changes, resulting in the breaking and reforming of transient HBs [14,15,16]. Transient HBs form free volume (FV) and localized stress in wood cell wall [17,18,19]. Under an external load, the formation of FV and localized stress accelerates the shear slip between the crystalline and amorphous phases in the cell wall [20,21]. In general, the MS effect is attributed to an unstable state in the wood cell wall under the external load when RH varies. When environmental conditions such as humidity and temperature change, the moisture gradient and stress gradient occur in the wood cell wall, which disturbs the equilibrium state of the molecular packing mode and creates localized stress in the wood cell wall [22,23]. The moisture gradient and stress gradient are presented as unstable state in the wood cell wall, which could explain the increasing damping when MC varied. To investigate the unstable state, creep tests under a static external load were used [23,24,25]. The dynamic mechanical approach is also suitable to this purpose, which reveals that the energy dissipation increases as a function of MC variation [26,27,28]. In our previous study [27], the unstable state caused by reducing MCs in wood was investigated, and it was demonstrated that greater unstable states occurred during quicker periods of MC variation. Compared to the desorption process, the increment of moisture during the sorption process plasticizes the wood polymer networks. In addition, absorbed moisture during the sorption process could create FV and localized stress, and theoretically cause the unstable state in wood cell walls. However, there are not many studies describing the unstable state during the moisture sorption process, especially detected under dynamic test. With the aim of investigating the time dependence of the viscoelasticity during sorption, and characterizing the unstable state of the cell wall, the effects of RH ramping rate and RH isohume level on frequency-dependent viscoelastic properties will be studied. The applicability of Boltzmann’s superposition principle will also be examined by simulating the viscoelastic properties during the sorption processes.

## 2. Materials and Methods

### 2.1. Materials

**Figure 1:**
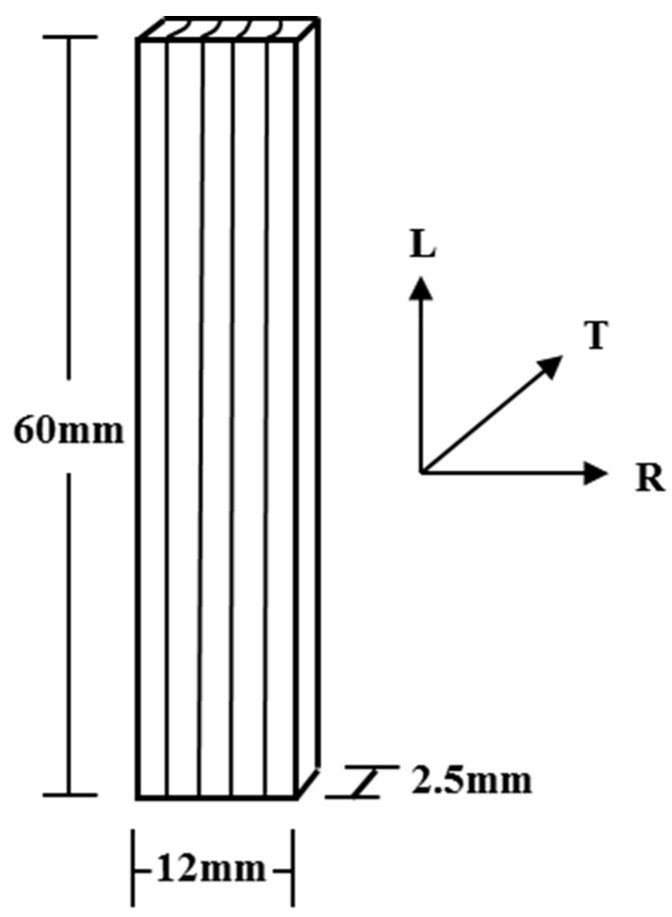
Schematic of wood specimen.

Wood specimens with a dimension of 60 longitudinal [L] × 12 radial [R] × 2.5 tangential [T] mm^3^ (Figure 1) were obtained from the outer region of air-dried heartwood (MC ≈ 12%) of Chinese fir (*Cunninghamia lanceolata* [Lamb.] Hook.). Clear specimens were cut successively from the same growth rings. All specimens were dried in a sealed container over P_2_O_5_ at 30 °C for more than nine weeks until a constant mass was achieved. The MC of dried specimens was approximately 0.6%. The raw density of specimens was 0.37 ± 0.02 g·cm^−3^. A total of 72 specimens were tested.

### 2.2. Measurements of the Viscoelasticity

**Figure 2:**
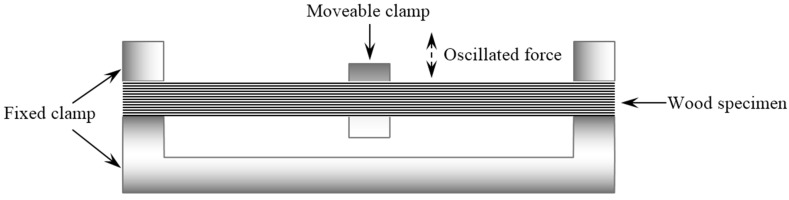
Schematic of dual-cantilever bending mode.

The dynamic mechanical analysis was performed with the instrument (TA Instruments, DMA Q800) equipped with a DMA-RH accessory, which is able to control the specimen environment in the range of 0% to 90% RH between 5 and 90 °C by modulating the flow rates of the dry nitrogen and saturated moisture. When the flow rate was completely dry nitrogen, the RH in the chamber was zero theoretically. In our previous studies, the actual lowest RH value cloud was close to zero. Therefore, the lowest RH value in this study was pre-set to zero with the neglected deviation. The viscoelasticity was determined at frequencies of 1 and 20 Hz. A dual-cantilever clamp with a distance of 35 mm was applied (Figure 2). The displacement amplitude created by the oscillated force was 15 μm. Specimens were clamped on the radial surfaces and bending occurred in the tangential direction. After being mounted on the clamp in the testing chamber, the specimens were kept under isohume conditions (30 °C, 0% RH) for 30 min prior to the actual viscoelastic measurements. For investigating the viscoelasticity during the sorption process, three operating modes (RH_ramp_, RH_isohume_, and RH_step_) were conducted. During the DMA test, a storage modulus *E*′, a loss modulus *E*″, and a loss factor tanδ (tanδ = *E*″/*E*′) were automatically calculated. *E*′ is the ability of the material to store energy, and represents the elastic part of the material. *E*″ is the viscous response of the material and is proportional to its dissipated energy.

#### 2.2.1. RH_ramp_ Mode

**Figure 3:**
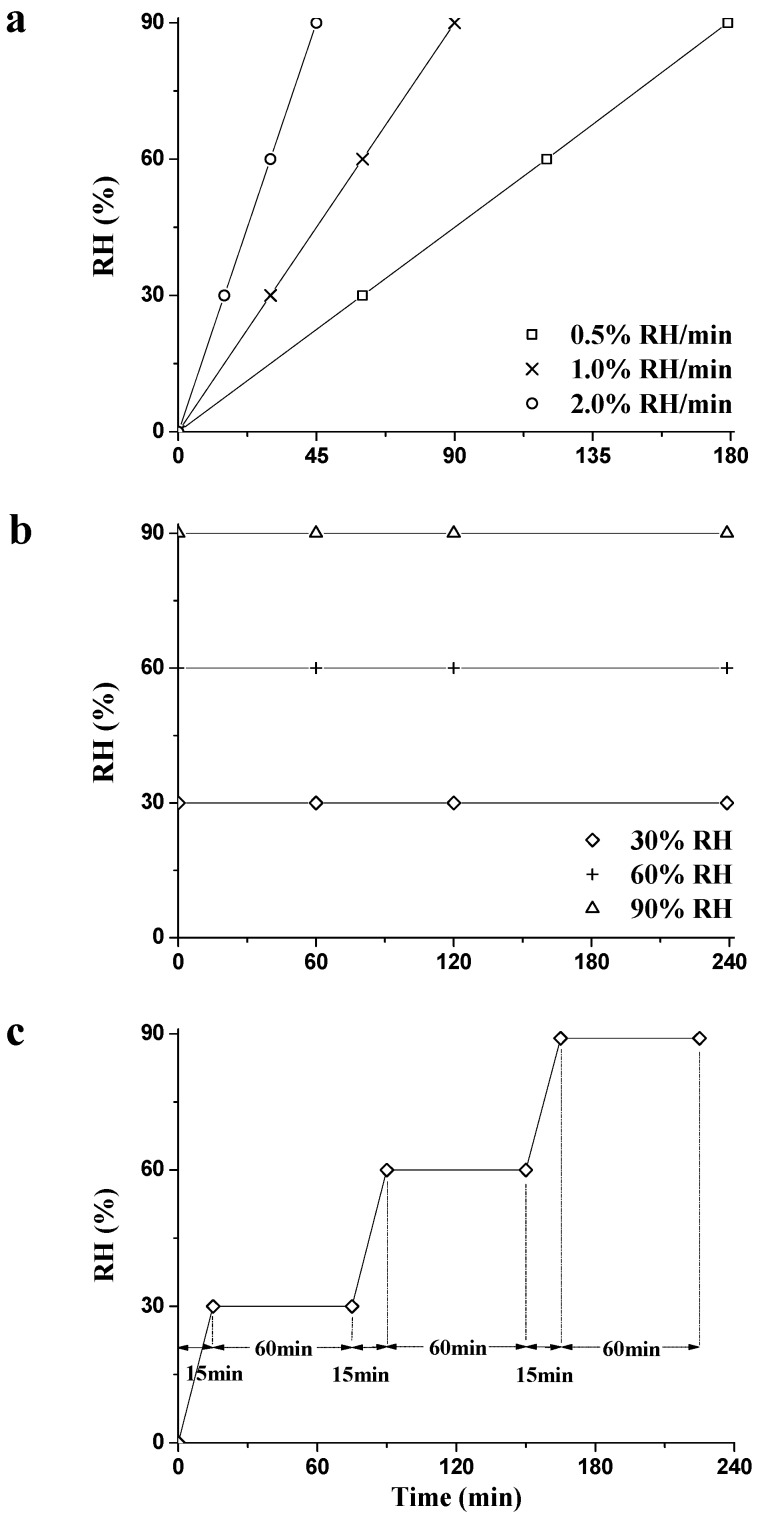
Outline of the experimental setup for the RH_ramp_ (**a**); RH_isohume_ (**b**); and RH_step_ mode (**c**).

RH in the chamber ramped up from 0% to 90% RH with ramping rates of 0.5%, 1.0%, or 2.0% RH/min, respectively. MC was determined by weighing specimens before and after each RH_ramp_ conditioning. To obtain the MC changing trend, some other specimens were also tested under the same RH_ramp_ condition, but only until the time points marked as symbols in Figure 3a. Three replicates for each test were performed and the results plotted in the figures are the average values of the three replicates.

#### 2.2.2. RH_isohume_ Mode

RH in the chamber was firstly adjusted from 0% to 30%, 60%, or 90% RH, with the ramping rate of 2.0% RH/min. Then, the isohume condition was kept for 240 min. MC was determined by weighing, as indicated above. Some specimens were also tested under the same RH_isohume_ condition, but only until the time points marked as symbols in Figure 3b. Three replicates for each test were performed and the results plotted in the figures are the average values of the three replicates.

#### 2.2.3. RH_step_ Mode

RH in the chamber was adjusted by a ramping rate of 2% RH/min from 0% to 30%, 60%, and 90% RH, successively. The isohume conditions were kept for 60 min. To acquire the MC changing trend, some specimens were also tested until reaching the RH_ramp_ or RH_isohume_ stages marked as symbols in Figure 3c [28]. Three replicates for each test were performed and the results plotted in the figures are the average values of the three replicates. 

### 2.3. Measurement of the Equilibrium Moisture Content (EMC)

Equilibrium moisture contents (EMCs) were reached under constant RH conditions (30%, 60%, and 90% RH) during the isothermal sorption tests, which was determined by means of a dynamic vapor sorption (DVS) Intrinsic apparatus (Surface Measurement Systems Ltd., London, UK). Specimens were prepared from wood chips (ca. 15 mg) after the samples were dried over P_2_O_5_. The preset RH was increased in steps in the preprogrammed sequence (0%, 30%, 60%, and 90% RH). The sorption processes were performed at a constant temperature of 30 °C in the whole RH range. Before moving to the next RH level, the instrument maintained the specimen at the constant RH until the weight changes per minute (d*m*/d*t*) were less than 0.002% per minute. Three replicates for the test were performed and the results plotted in the figures are the average values of the three replicates.

## 3. Results and Discussion

### 3.1. Viscoelasticity under RH_ramp_ Mode

**Figure 4:**
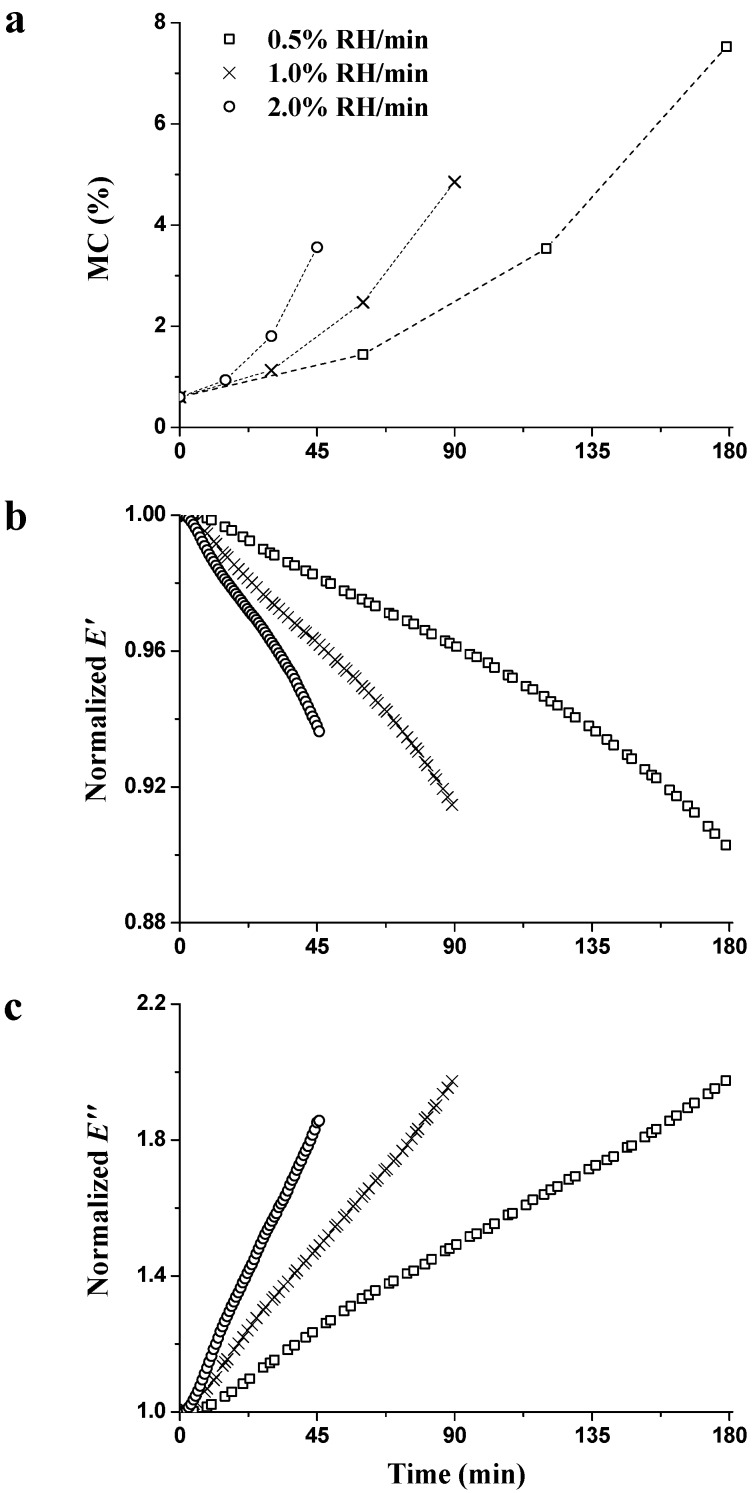
Changes of wood MC (**a**); n*E*′ (**b**); and n*E*″ (**c**) during the RH_ramp_ period (0% → 90% RH) with different RH ramping rates measured at 1 Hz.

Figure 4 shows the time dependence of moisture sorption (Figure 4a), which is related to the normalized (n) data concerning n*E*′ (Figure 4b) and n*E*″ (Figure 4c) during the RH_ramp_ period (0% → 90% RH) with different ramping rates measured at 1 Hz. The normalized data are calculated:

n*E*′(t) = *E*′(t)/*E*′_0_, (1)

n*E*″(t) = *E*″(t)/*E*″_0_, (2)

where the lower case ‘0’ designates the corresponding data at the beginning of the RH_ramp_ period. 

Higher MC values were achieved as a function of time, regardless of the ramping rate (Figure 4a). At the end of the RH_ramp_ period, MC increased by 7.5%, 4.9% and 3.6% at 0.5%, 1.0%, and 2.0% RH/min, respectively. The higher the RH ramping rate was, the lower the final MC value was obtained. Wood is a hygroscopic material. Moisture is attracted to the surfaces of the test specimen and pores, and absorbed within the wood cell walls. The moisture sorption quantity is determined by the number of polar groups (hydroxyl) in wood’s main chemical components, while the sorption rate is influenced by the moisture diffusion rate in the specimen. The moisture diffusion rate is closely related to the structural anatomy of the wood. When moisture penetrates from the specimen shell to the core, the diffusion rate lags behind the RH changes due to the large resistance donated by the lumen–cell wall interface and the interior of the cell wall [29]. In addition, the kinetic sorption was linked to the glass transition of hemicellulose, which allowed for accommodation of more water molecules within the wood cell walls [10]. Lower ramping rate meant longer sorption time within the same RH region, i.e., the longer the sorption time, the higher the final MC was during the transient stage. 

During the whole RH_ramp_ period, quasi-linear decrement of n*E*′ and increment of n*E*″ were observed in Figure 4b,c. At the end of the RH_ramp_ period, n*E*′ were 0.89, 0.91, and 0.93 at 0.5%, 1.0%, and 2.0% RH/min ramping rates, respectively. n*E*″ were 2.00, 2.00, and 1.86 at 0.5%, 1.0%, and 2.0% RH/min ramping rates, respectively. The decreasing normalized *E*′ and the increasing normalized *E*″ are attributed to the increase in MC. Water acts as a plasticizer to affect the wood stiffness and soften hemicellulose [10,30]. When penetrating into the wood cell wall, water molecules break HBs within the polymers and form HBs between water molecular and amorphous components (hemicellulose, lignin, and paracrystalline cellulose). The plasticization of the amorphous polymers enhances the flexibility of the polymer network [31]. The increasing degree of n*E*″ was obviously higher than the decreasing degree of n*E*′, regardless of the ramping rate. Takahashi et al. confirmed that the decrease in elasticity is not as great as the increase in damping during the sorption process [32]. Since cellulose, hemicellulose, and lignin have different absorbability, the extents of hygro-expansion vary within the wood cell walls, providing the shear slip between cellulose and matrix, and leading to a large energy dissipation [21,24].

**Figure 5:**
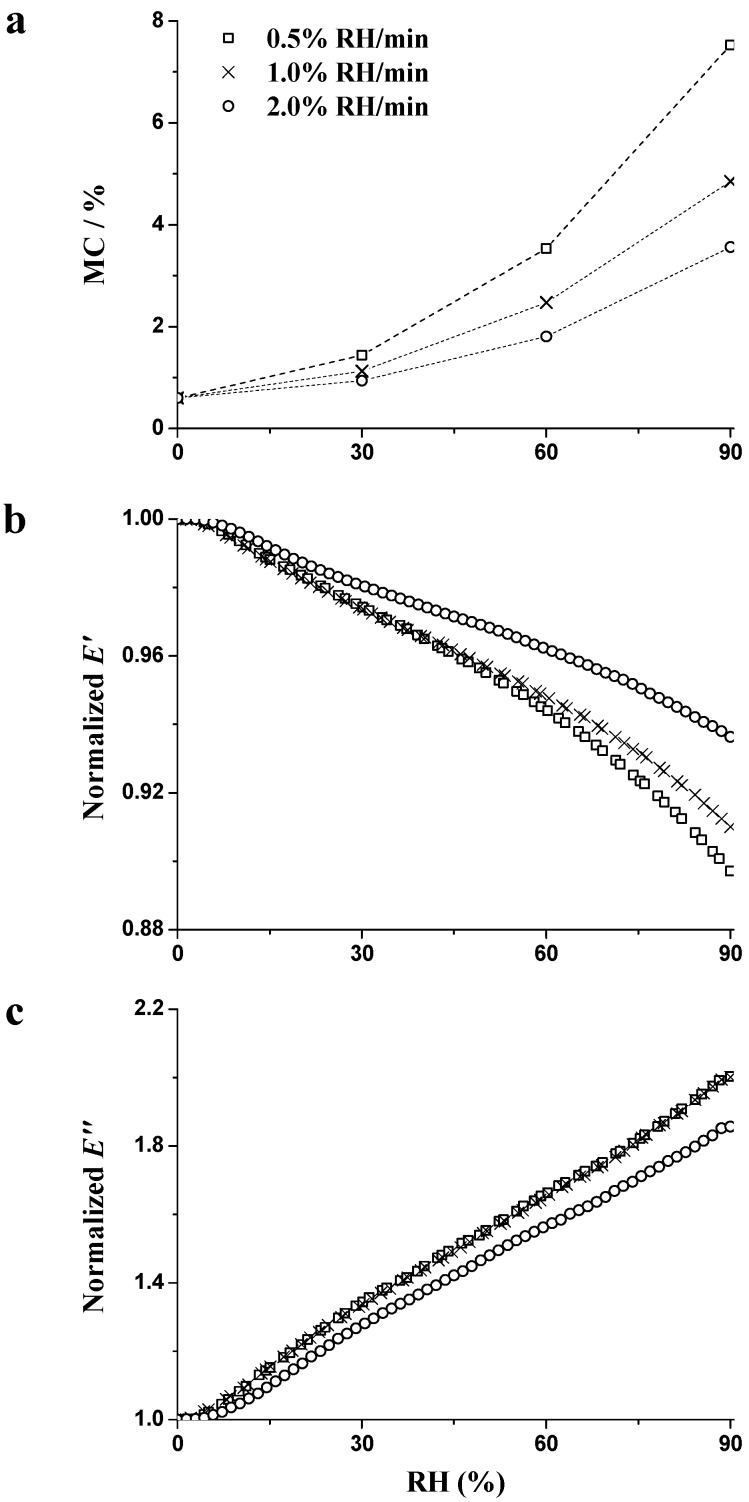
Influence of RH on the MC (**a**); normalized *E*′ (**b**); and normalized *E*″ (**c**) during the RH_ramp_ period (0% → 90% RH) with different RH ramping rates.

The constant ramping rates of RH made it possible to establish the relationships between RH and viscoelasticity. In Figure 5, the MC and viscoelasticity as a function of RH are presented with various RH ramping rates. Not only MC, but also the higher increasing rate of MC was observed at a higher RH level, regardless of the ramping rate (Figure 5a). This is because the new adsorptive sites produce and further increase the amounts of sorption water.

Changes of n*E*″ were almost the same with increasing RH when the ramping rates were 0.5% and 1.0% RH/min (Figure 5c), although MC and n*E*′ showed discrepancies of behavior at these two ramping rates (Figure 5a,b). This result may be associated with the existence of an unstable state in the cell wall. The unstable state is the representation of the moisture gradient and stress gradient when RH varies. When penetrating into the wood cell wall, moisture molecules not only break HBs within wood polymer, but also associate with the promotion of FV in the wood cell wall for the motions of surrounding polymer substances. The FV in the wood cell wall forms localized stress and disturbs the equilibrium packing of polymer molecules, resulting in more energy dissipation [17,23]. The changes of n*E*″ could be explained by the double-effect of plasticization effect and unstable state. The parameters of |∆n*E*′/∆MC| and |∆n*E*″/∆MC| were used to evaluate the double-effect and calculated as

|∆n*E*′/∆MC| = |(n*E*′_i_ − n*E*′_0_)/(MC_i_ − MC_0_)| (3)

|∆n*E*″/∆MC| = |(n*E*″_i_ − n*E*″_0_)/(MC_i_ − MC_0_)|, (4)

where the subscript ‘i’ designates the corresponding data when a predefined RH of 30%, 60%, or 90% is reached, and the subscript ‘0’ designates the corresponding data at the beginning of the RH_ramp_ period. As seen in Figure 6, higher |∆n*E*′/∆MC| and |∆n*E*″/∆MC| were found at lower RH regardless of the ramping rate. On the one hand, these results are related to the varied plasticization effect among different water layers (monomolecular or polymolecular water layer) within the wood cell wall [33]. The plasticization effect on chain segments of wood polymers is likely to be much greater for the monomolecular water layer, and subsequently less for each additional polymolecular water layer [5]. On the other hand, the influence of FV reduces with the increasing MC, i.e., the unstable state diminishes as a function of sorption time [24]. In addition, higher |∆n*E*′/∆MC| and |∆n*E*″/∆MC| were obtained at higher ramping rates, which confirms that higher ramping rates produce greater destabilization during the sorption process [24,27,34,35].

**Figure 7:**
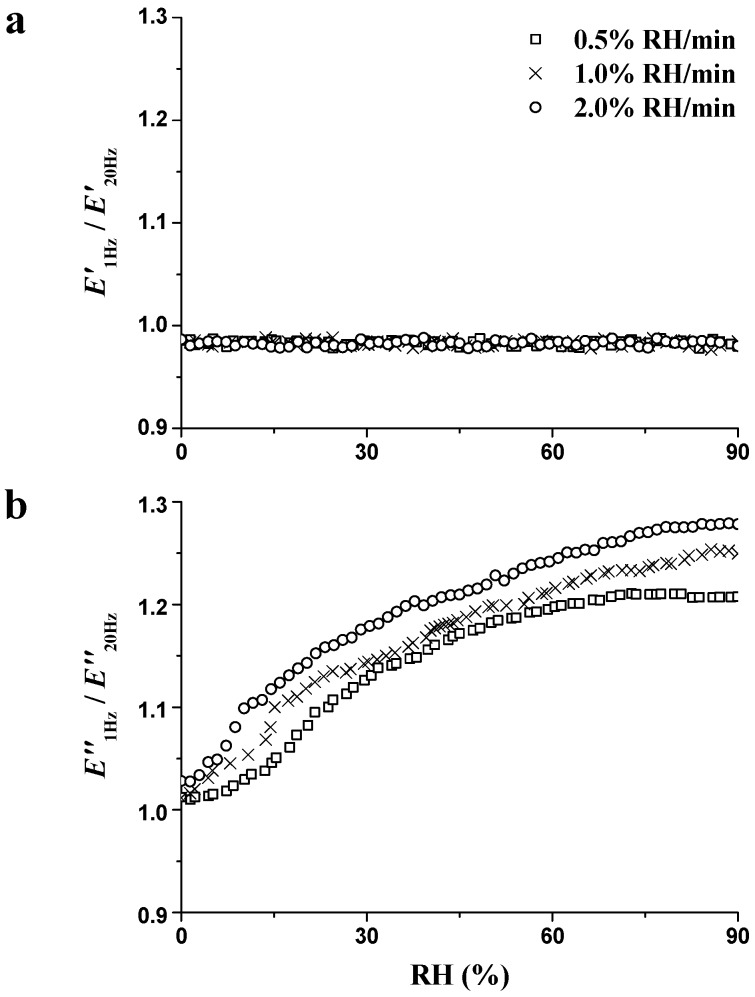
Influence of RH on *E*′_1Hz_/*E*′_20Hz_ (**a**) and *E*″_1Hz_/*E*″_20Hz_ (**b**) during the RH_ramp_ period (0% → 90% RH) with different RH ramping rates.

In our previous study, it was proposed that changes in the *E*″ ratio at different frequencies were proposed as an effective method for directly evaluating the unstable state induced by the varying MC [27]. The ratios of *E*″ at 1 and 20 Hz (*E*″_1Hz_/*E*″_20Hz_) during the whole RH_ramp_ period were calculated and are presented in Figure 7b, combined with the *E*′ ratios at 1 and 20 Hz (*E*′_1Hz_/*E*′_20Hz_) in Figure 7a. *E*′_1Hz_/*E*′_20Hz_ ranged at 0.98 without any obvious variation, proving that the stiffness is not influenced by the unstable state at different frequencies. When the stiffness of wood is determined, especially along the longitudinal direction, the most attention is given to cellulose. The rationale is that the cellulose with its highly arranged crystalline structure is the stiff, reinforcing material in the wood cell wall [36]. The most recent analysis estimated that the elastic modulus in the longitudinal direction (*E*x) for the cellulose crystalline region ranged between 120 and 170 GPa [37]. It is found that *E*x basically does not vary with the increasing MC because the moisture is only absorbed on the microfibril surfaces and does not penetrate into the interior spaces. Therefore, the stiffness is not affected by the unstable state.

*E*″_1Hz_/*E*″_20Hz_ (Figure 7b) increased with the increase in RH, indicating the presence of an unstable state and the energy dissipation with the RH increment. The changes of *E*″ ratios at 1 and 20 Hz could be caused by that lower frequency, which allows a more complete evolution of the unstable state [38]. Hence the increase in *E*″_1Hz_/*E*″_20Hz_ would be observed when the unstable state is more pronounced. Furthermore, at the end of the RH_ramp_ period, *E*″_1Hz_/*E*″_20Hz_ was 1.21, 1.25, and 1.28 at ramping rates of 0.5%, 1.0%, and 2.0% RH/min, respectively. Greater values of *E*″_1Hz_/*E*″_20Hz_ were found at higher ramping rates, which confirmed the increasing destabilization effect and more energy dissipation.

### 3.2. Viscoelasticity under RH_isohume_ Mode

**Figure 8:**
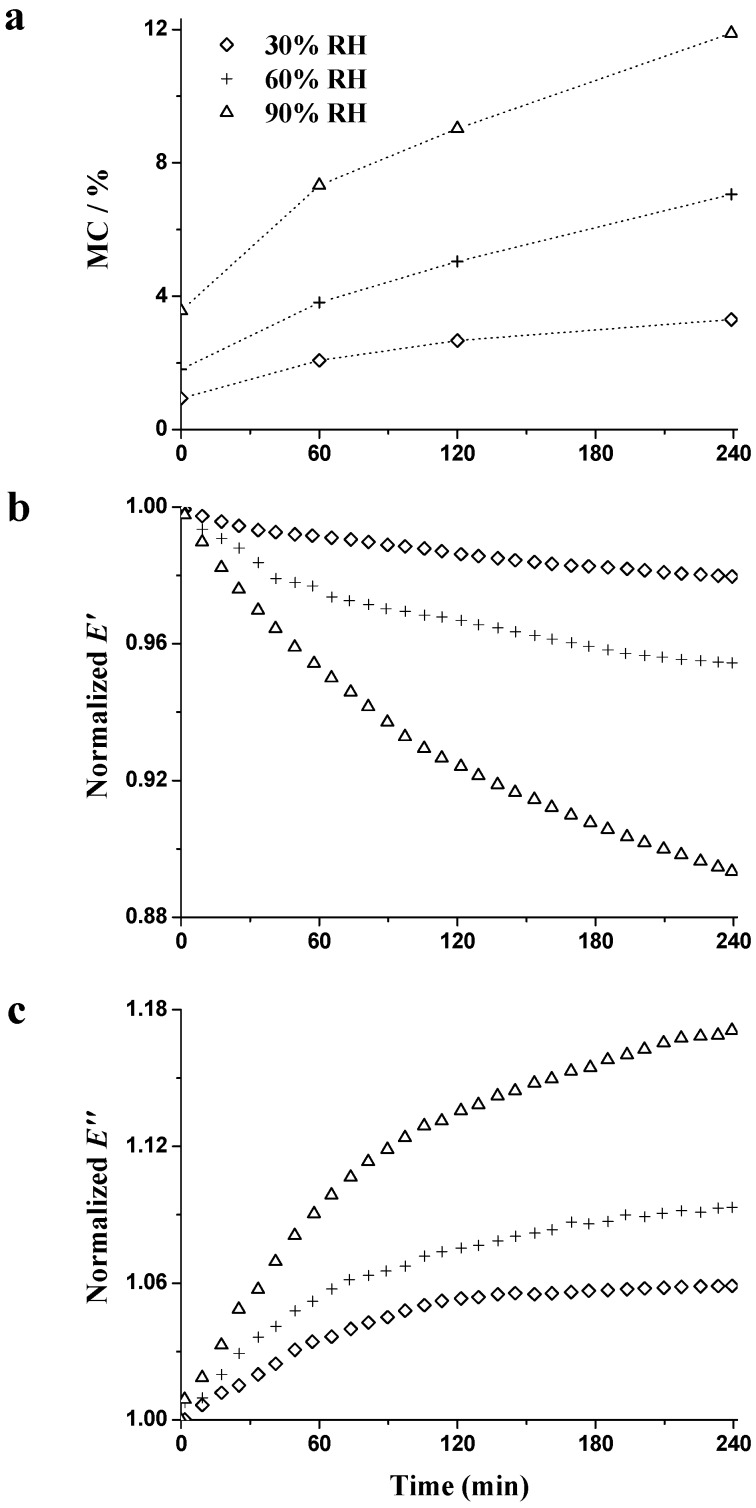
Changes of the MC (**a**); normalized *E*′ (**b**); and normalized *E*″ (**c**) during the RH_isohume_ periods (30%, 60%, and 90% RH) measured at 1 Hz.

**Figure 9:**
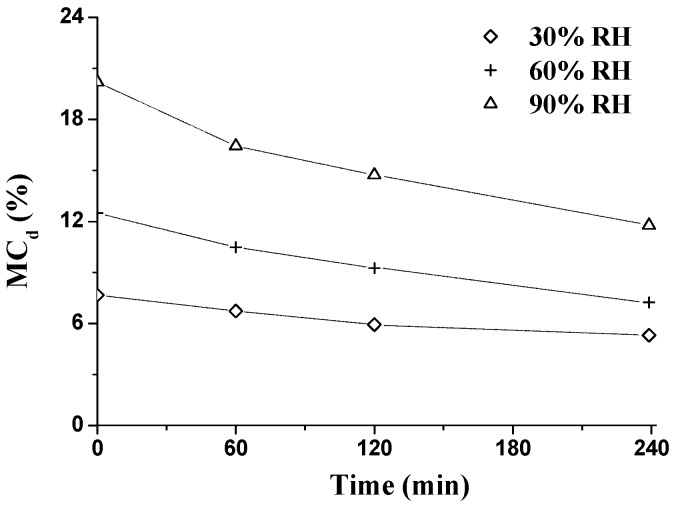
Changes of MC_d_ (difference value of MC and EMC) during the RH_isohume_ periods (30%, 60%, and 90% RH).

The time-dependent nature of wood moisture sorption, n*E*′ and n*E*″, during the RH_isohume_ period (30%, 60%, and 90% RH) is shown in Figure 8. During the RH_isohume_ period, a lower increasing rate of MC was found as a function of time, showing that the wood is approaching EMC during the isohume exposure period. The higher the RH level, the higher and faster increase in MC was found. After the RH_isohume_ period of 240 min, the final values of MC were 3.4%, 7.1%, and 12.1% at 30%, 60%, and 90% RH, respectively (Figure 8a). Wood gains moisture from the surrounding atmosphere and approaches EMC. Through the DVS tests, EMC at 30%, 60%, and 90% RH is 8.0%, 13.7%, and 23.2%, respectively. In Figure 9, MC_d_ (the difference value of MC during the RH_isohume_ period and EMC at 30%, 60%, and 90% RH) was calculated as

MC_d_ = EMC_a_ − MC_i_, (5)

where the subscript ‘a’ represents the EMC value at 30%, 60%, or 90% RH, by DVS tests, and the subscript ‘i’ designates the corresponding MC at each RH_isohume_ time point (60, 120, or 240 min). Regardless of the RH_isohume_ level, MC_d_ decreased as a function of time. Within the same isohume time, the higher the RH level, the more pronounced decreasing rate was obtained, i.e., the higher the sorption rate. The sorption rate is controlled by the external resistance from the boundary layer and the internal resistance from the wood cell walls. Avramidis and Siau found that the external resistance decreases with the increasing RH, confirming that quicker sorption rates could be observed at higher RH levels [39].

n*E′* (Figure 8b) decreased and n*E*″ (Figure 8c) increased during all three RH_isohume_ periods (30%, 60%, and 90% RH). The higher the RH level, the lower the n*E*′ and the higher the n*E*″ observed. These results confirm the plasticization effect of water, which enhanced the flexibility of the polymer network [31]. Compared to n*E*′, greater changes of n*E*″ were found at all three RH_isohume_ levels, which is proved by the large change in energy dissipation owing to the shear slip between the cellulose and matrix [21,24,40]. The changes of |∆n*E*′/∆MC| and |∆n*E*″/∆MC| were calculated as

|∆n*E*′/∆MC| = |(n*E*′_i_ − n*E*′_0_)/(MC_i_ − MC_0_)| (6)

|∆n*E*″/∆MC| = |(n*E*″_i_ − n*E*″_0_)/(MC_i_ − MC_0_)|, (7)

where the subscript ‘i’ designates the corresponding data at each RH_isohume_ time point (60, 120, or 240 min) while the subscript ‘0’ is for the data obtained at the beginning of the RH_isohume_ period.

**Figure 10:**
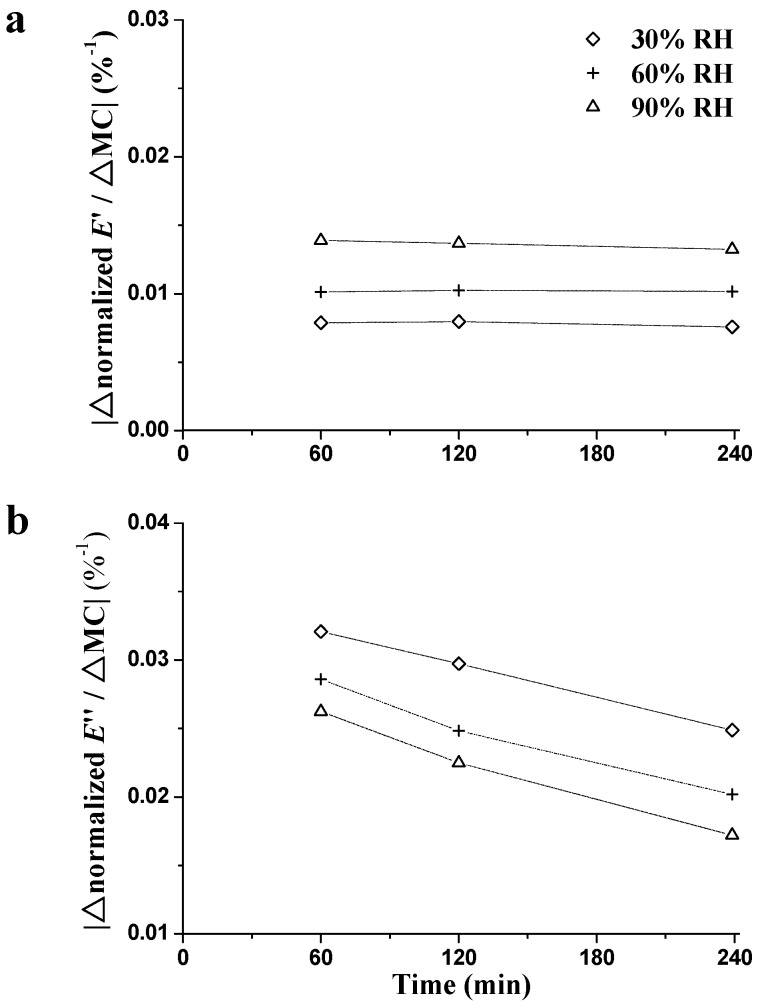
Influence of isohume time on the absolute changes of normalized *E*′ (**a**) and normalized *E*″ (**b**) per unit change in MC during the RH_isohume_ periods (30%, 60%, and 90% RH).

These values are plotted as a function of time in Figure 10. The values of |∆nE′/∆MC| (Figure 10a) were basically constant, and ranged by about 0.01 at all three RH_isohume_ levels. The basically constant values of |∆n*E*′/∆MC| explain why the wood stiffness has a linear relationship with MC under RH_isohume_ conditions [41,42]. A linear decrease of |∆n*E*″/∆MC| (Figure 10b) was observed regardless of RH_isohume_ level. The lower values of |∆n*E*″/∆MC| could be observed as a function of sorption time, illustrating that |∆n*E*″/∆MC| decreased with the increasing MC. These results can probably be attributed to two aspects: (1) more rapid sorption is associated with greater changes in damping, especially early in the RH_isohume_ period; and (2) during the RH_isohume_ period, the unstable state of wood cell wall is mitigated and polymers can be stabilized because of the reorientation of molecular chains [24,27,34,35].

**Figure 11:**
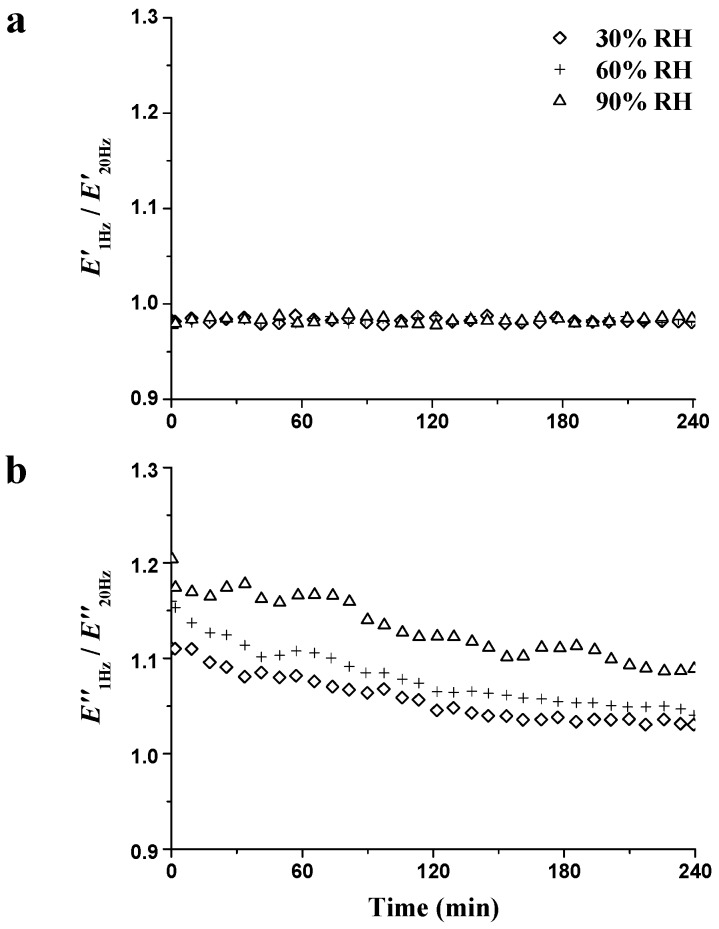
Changes of *E*′_1Hz_/*E*′_20Hz_ (**a**) and *E*″_1Hz_/*E*″_20Hz_ (**b**) during the RH_isohume_ periods (30%, 60%, and 90% RH).

**Figure 12:**
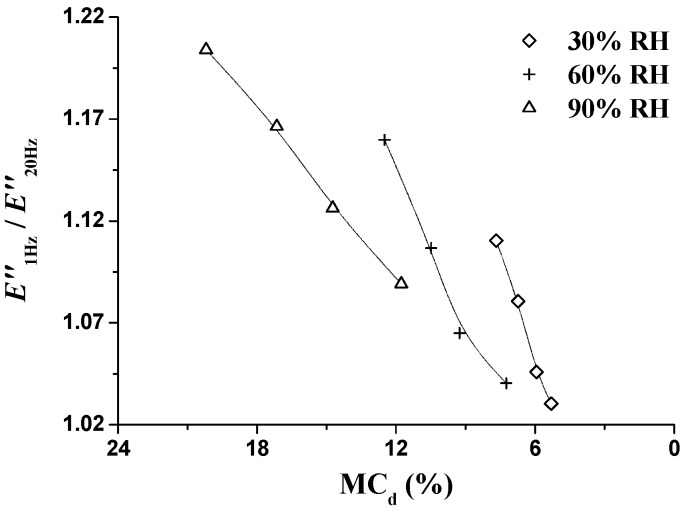
Relations of *E*″_1Hz_/*E*″_20Hz_ and MC_d_ during the RH_isohume_ periods (30%, 60%, and 90% RH).

In order to analyze the unstable state during the RH_isohume_ period, the values of *E*′_1Hz_/*E*′_20Hz_ and *E″*_1Hz_/*E*″_20Hz_ were also calculated and are presented in Figure 11. *E*′_1Hz_/*E*′_20Hz_ (Figure 11a) remained at 0.98 with no obvious variation, which is consistent with the results during the RH_ramp_ period. *E*″_1Hz_/*E*″_20Hz_ (Figure 11b) decreased with the RH_isohume_ time regardless of RH level, indicating the diminishing energy dissipation and mitigating the unstable state. At the end of the RH_isohume_ period, *E*″_1Hz_/*E*″_20Hz_ decreased by 7.2%, 9.5%, and 10.3% at the RH_isohume_ level of 30%, 60%, and 90% RH, respectively. Much reduction in *E*″_1Hz_/*E*″_20Hz_ at higher RH_isohume_ indicates a rapid recovery of the unstable state. To investigate the relationships between unstable state and MC_d_, the values of *E*″_1Hz_/*E*″_20Hz_ at RH_isohume_ time points (60, 120, and 240 min) were plotted as a function of MC_d_, as shown in Figure 12. *E*″_1Hz_/*E*″_20Hz_ quasi-linearly decreased with the decreasing MC_d_ at all three RH_isohume_ levels. The decreasing in *E*″_1Hz_/*E*″_20Hz_ represents that the unstable state diminishes when MC approaches the equilibrium value, i.e., the EMC value. In addition, the higher the isohume RH level, the greater the reduction in *E*″_1Hz_/*E*″_20Hz_ observed, showing that a higher RH level provides more mitigation of the unstable state during the RH_isohume_ period.

### 3.3. Viscoelasticity under RH_step_ Mode

**Figure 13:**
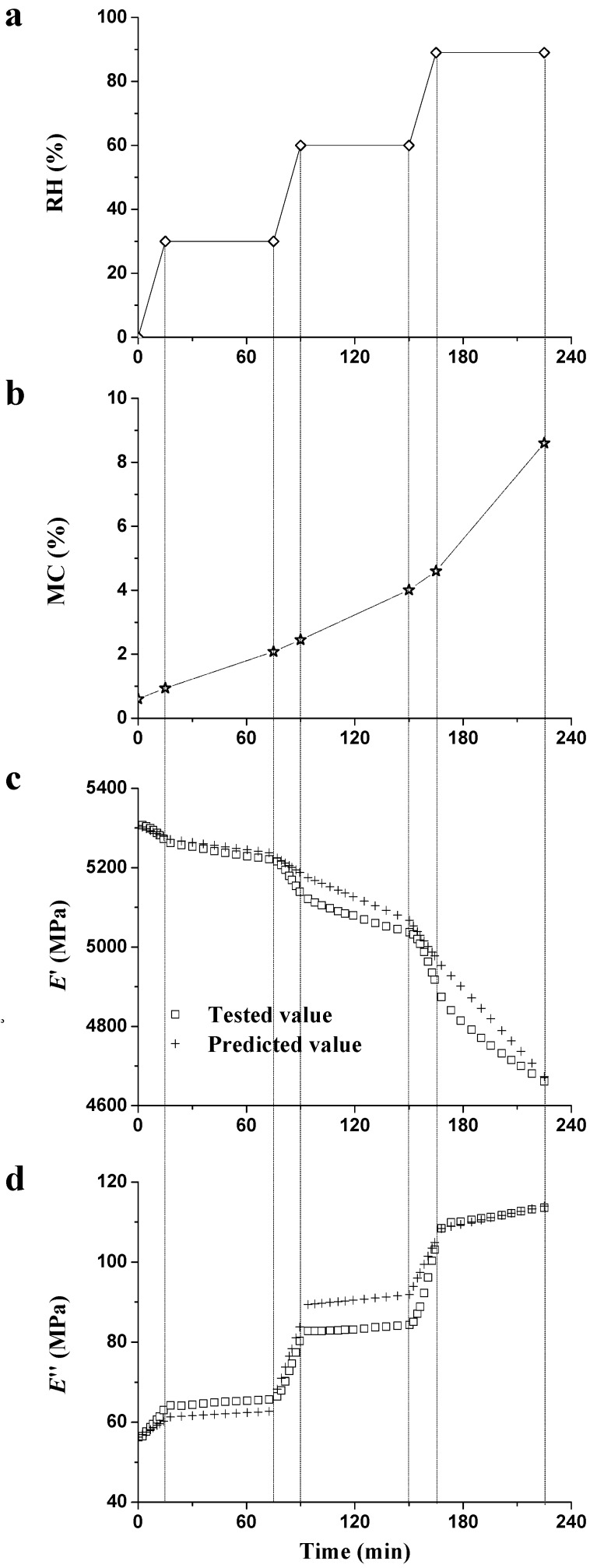
Changes of the RH (**a**); MC (**b**); normalized *E*′ (**c**); and normalized *E*″ (**d**) during the RH_step_ period (0% → 30% → 60% → 90% RH).

The changes of ambient RH (a), wood MC (b), *E*′ (c), and *E*″ (d) are presented in Figure 13. The changes of stiffness (Figure 13c) and damping (Figure 13d) during the RH_step_ period are related to the plasticization and the unstable state effects [28]: The plasticization effect of moisture molecules causes decreasing stiffness and increasing damping, no matter whether RH is varied or constant. Concerning the unstable state effect, the varied RH, as a driving force, accelerates the sorption of water molecules on the specimen surface and aggravates the moisture gradient from the specimen shell to core [29]. On the other hand, localized stresses occur when external stresses are concentrated in the more moisture-sensitive load-bearing elements, such as the matrix, which would further aggravate the localized stress-driven (or heterogeneity-driven) unstable state effect [43].

In order to simulate viscoelasticity during the RH_step_ period, Boltzmann’s superposition principle was used to estimate in each RH_ramp_ and RH_isohume_ stage. Zhuoping confirmed that the rheology properties of wood could be simulated conveniently by the application of the generalized Boltzmann’s superposition principle [44]. The summations of the viscoelasticity in each stage were expressed as:

(8) $$E^{\prime }={E^{\prime }}_{0}+\sum_{i}^{n}E^{\prime }(\Delta {\mathrm{MC}}_{i},\Delta t_{i})$$

(9) $$E^{\prime \prime }={E^{\prime \prime }}_{0}+\sum_{i}^{n}E^{\prime \prime }(\Delta {\mathrm{MC}}_{i},\Delta t_{i}),$$

where *E*′_0_ and *E*″_0_ are the initial values in the RH_step_ period. ∆t equals 15 or 60 min for the time of RH_ramp_ or RH_isohume_ stage, respectively. When MC increased continuously, Equations (8) and (9) could be depicted by the integrals:

(10) $$E^{\prime }={E^{\prime }}_{0}+\int_{o}^{t}\frac{\partial E^{\prime }}{\partial MC\cdot \partial t}dMCdt$$

(11) $$E^{\prime \prime }={E^{\prime \prime }}_{0}+\int_{o}^{t}\frac{\partial E^{\prime \prime }}{\partial MC\cdot \partial t}dMCdt.$$

Based on the regressions of *E*′, *E*″, and MC during the RH_ramp_ and RH_isohume_ stages, the predicted values of *E*′ and *E*″ are presented in Figure 13c,d, together with the tested values. In Figure 13c, the discrepancy of predicted and tested value of stiffness could be obviously observed when RH was up to 60%; meanwhile, the prediction of damping was not well fitted when RH remained constant at 30% or 60% RH (Figure 13d). The deviations of stiffness and damping were probably associated with the varied unstable state effect when RH changed or remained constant, which merits further investigation.

## 4. Conclusions

The influence of RH on the viscoelastic properties of the Chinese fir during sorption processes was investigated by the RH_ramp_, RH_isohume_, and RH_step_ modes. The conclusions of this study are as follows:

(1) The changes of *E*′ and *E*″ during moisture sorption processes are attributed to the water plasticization effect and the unstable state in wood cell walls. The unstable state is related to the breaking of hydrogen bonds within the polymer network, and the formation of free volume in cell walls.
(2) Higher ramping rates aggravated the unstable state during the RH_ramp_ period, and higher constant RH levels provided more recovery of the unstable state during the RH_isohume_ period. During the sorption process, the more recovery of unstable state was observed when MC was approaching EMC.
(3) Changes of viscoelasticity are associated with whether RH varied or remained constant. The varied RH, as a driving force, aggravated the internal stress gradients and accelerated the unstable state. The application of Boltzmann’s superposition principle is an approach to simulate the viscoelasticity in an in-depth investigation.

## Figures and Tables

**Figure 6:**
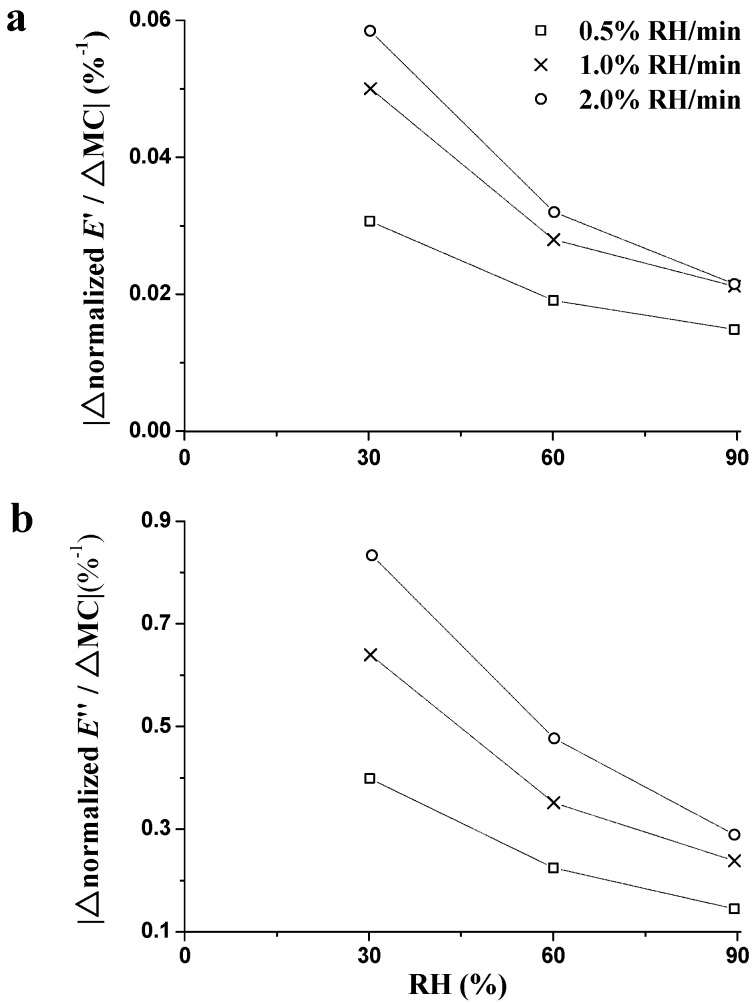
Influence of RH on the absolute changes of normalized *E*′ (**a**) and normalized *E*″ (**b**) per unit change in MC with different RH ramping rates.
